# Quality of health care for patients with coronary heart disease and comorbid mental disorders: a prospective cohort study

**DOI:** 10.1186/s40359-024-01693-x

**Published:** 2024-05-23

**Authors:** Laura Nordmeyer, Charlotte Leikert, Lena Sannemann, Kai Keller, Christin Leminski, Adriana Meixner, Samia Peltzer, Belinda Werner, Ludwig Kuntz, Holger Pfaff, Frank Schulz-Nieswandt, Frank Jessen, Christian Albus, Cologne Research and Development Network (CoRe-Net) Study Group, Ingo Meyer, Nadine Scholten, Stephanie Stock, Julia Strupp, Raymond Voltz

**Affiliations:** 1 Department of Psychosomatics and Psychotherapy, Faculty of Medicine and Cologne University Hospital, University of Cologne, Cologne, Germany; 2 Department of Psychiatry and Psychotherapy, Faculty of Medicine and Cologne University Hospital, University of Cologne, Cologne, Germany; 3 Chair of Quality Development and Evaluation in Rehabilitation, Faculty of Human Sciences & Faculty of Medicine and University Hospital Cologne, Institute for Medical Sociology, Health Services Research and Rehabilitation Science, University of Cologne, Cologne, Germany; 4 Department of Business Administration and Health Care Management, Faculty of Management, Economics and Social Sciences, University of Cologne, Cologne, Germany; 5 Faculty of Management, Economics and Social Sciences, Institute of Sociology and Social Psychology (ISS), University of Cologne, Cologne, Germany; 6 Centre for Health Services Research Cologne (ZVFK), University of Cologne, Cologne, Germany; 7 German Center for Neurodegenerative Diseases (DZNE), Bonn, Germany; 8 Excellence Cluster on Cellular Stress Responses in Aging-Associated Diseases (CECAD), University of Cologne, Cologne, Germany

**Keywords:** Coronary heart disease, Mental disorders, Delivery of health care, Primary care, Prospective studies

## Abstract

**Background:**

Coronary heart disease (CHD) is often associated with mental disorders (MDs). Comorbid MDs reduce the quality of life and increase cardiac morbidity and mortality. Nevertheless, there is little and inconsistent research on the management of MDs in CHD patients. To bridge this gap, this study aims to gain insight into the long-term course of MD-related health care for patients with CHD, in order to identify opportunities for care improvement.

**Methods:**

In this prospective cohort study, we investigated whether CHD patients with or without expert-rated MD at baseline (*N* = 364) received different MD-related health care from either their general practitioner (GP) or cardiologist at follow-up,* M* = 2.7 [2.0–4.0] years later. In the follow-up assessment, *N* = 131 CHD patients participated and received questionnaires capturing sociodemographic, mental health, and MD-related health care characteristics. Descriptive statistics, t-tests and chi-squared tests were used for analyses.

**Results:**

We found significant differences in MD-related health care. CHD patients with MD were more likely to be examined psychologically/psychiatrically (MD 55.9%, non-MD 16.7%, *p* = < .001) and diagnosed with MD (MD 55.9%, non-MD 13.5%,* p* = .020) by their GP or cardiologist. Recommendations for and responses to requests for psychotherapy were more likely in MD patients compared to non-MD patients (MD 38.7%, non-MD 11.8%, *p* = .012 and MD 38.5%, non-MD 11.8%,* p* = .031, respectively). No significant differences were found concerning physicians’ active demand for patients’ mental health, referral to a specialist for additional diagnostics, provision of information about the diagnosed MD and further treatment options, response to the patients’ request for psychopharmacotherapy, help received in finding psychotherapy or psychopharmacotherapy, and actual receipt of these treatments.

**Conclusions:**

The results indicate differences in MD-related health care of CHD patients with and without comorbid MD. However, they still highlight the need to further encourage primary care physicians treating CHD to adequately address MDs, provide further diagnostics, support, and information to affected patients. To address this, physicians may benefit from awareness training on the association between CHD and MDs and on appropriate communication with MD patients.

**Trial registration:**

German clinical trials register (Deutsches Register Klinischer Studien, DRKS) Registration Number: ID DRKS00022154, date of registration: 02.11.2021.

## Background

Coronary heart disease (CHD) represents one of the largest disease burdens worldwide and is one of the leading causes of death [1]. Mental disorders (MDs) also contribute significantly to the global burden of disease [2]. At the same time, there is a relevant comorbidity of MD and CHD and the risk of developing an MD increases with existing CHD [3–5]. The 12-month prevalence of MDs in CHD patients is approximately 40% [6]. Studies also indicate that MD is associated with an increased risk of developing CHD and an unfavorable disease course. For example, depression is considered an independent risk factor for CHD incidence, higher cardiac morbidity, and mortality [7–9]. These findings are comparable for anxiety disorders [10, 11], schizophrenia and bipolar disorders [7].

Guidelines recommend regular screenings for MD [6, 12] and suggest specific treatment for comorbid MD in CHD, such as psychotherapeutic or psychopharmacological treatment [6]. However, screening and treatment appear to be inadequately implemented in routine care [13]. There seems to be a lack of knowledge among physicians regarding the cardiovascular disease guidelines [14] and the role of MDs like depression in CHD [15]. To date, depression in CHD remains largely under-recognised and under-treated [16–18]. Findings from a study by Peltzer et al. [19] highlighted that only half of the CHD patients who were diagnosed with MD in their study received a corresponding diagnosis from their treating physician and in addition, psychological difficulties were insufficiently addressed. Shortcomings regarding treatment recommendations were evident and only a marginal proportion of patients received psychotherapeutic/psychiatric treatment [19].

To date, research on the quality of health care and treatment trajectories of patients with CHD and comorbid MD has been limited and inconsistent. For instance, there is one study showing that health care of comorbid depression is treated well in patients with coronary artery disease, while there are shortcomings in the diagnosis and treatment of comorbid anxiety disorders [20]. In contrast, other studies show that there are also deficits in depression management [17, 18], in the implementation of treatment recommendations and in the diagnosis and treatment of MDs in general [19]. Therefore, we aimed to gain deeper insights into the treatment of CHD patients with MD in order to provide an overview of the current state of CHD care. This will allow the identification of care gaps in this vulnerable population and serve as a basis to identify opportunities for improvement in both detection and treatment of MDs, which might ultimately lead to an improvement of care in CHD patients.

In this study, we conducted a follow-up survey of CHD patients with and without expert-rated comorbid MD at baseline to assess differences in reported MD-related health care provided by a general practitioner (GP) or cardiologist at follow-up. Based on previous research showing insufficient guideline adherence [18, 19], we hypothesised that MD-related health care would not differ between CHD patients with and without MD for most aspects of care measured in this study.

## Methods

### Procedure and participants

This prospective cohort study is based on a project on mental and cognitive disorders in patients with coronary heart disease (MenDis-CHD), which is part of the interdisciplinary Cologne Research and Development Network (CoRe-Net). CoRe-Net is a competence network of practice and research for the model region of Cologne [21], funded by the German Federal Ministry of Education and Research (BMBF). The MenDis-CHD protocol has been approved by the Ethics Commission of the Faculty of Medicine of Cologne University (ID 20-1471) and is registered at the German Clinical Trials Register (ID DRKS00022154). The study was conducted in accordance with the Declaration of Helsinki, the General Data Protection Regulation (GDPR) and national data protection laws.

The MenDis-CHD I study [19, 22, 23] serves as a baseline sample from which participants were recruited for the follow-up survey. Inclusion criteria were equivalent to the first enrollment: Age ≥ 18 years, angiographically documented CHD after stable angina pectoris, acute coronary syndromes, percutaneous coronary intervention or bypass surgery and sufficient knowledge of German. Exclusion criteria were medically diagnosed severe or instable physical and/or psychological conditions (e.g., severe heart failure according to the assessment of the treating clinician, unstable angina pectoris, cancer, delirium, moderate to severe dementia, or acute suicidality). Patients were initially recruited between January 2018 and March 2019. We used cluster sampling with two hospitals, two rehabilitation clinics, and three cardiology practices in Cologne, Germany, as sampling units. As in one-stage cluster sampling, all eligible patients in the selected sampling units were included in the study if they wished to participate and met the inclusion criteria. Patients who had agreed to be contacted again for secondary study parts were sent a set of questionnaires to complete at home and return to the study team.

**Fig. 1:**
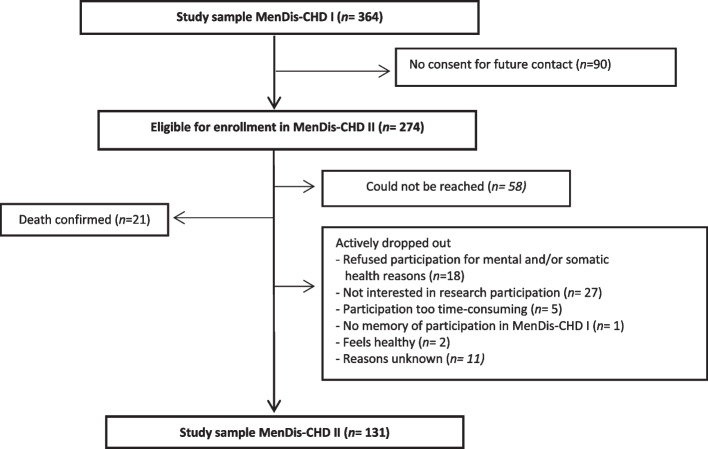
Study flowchart of participants from baseline to follow-up

The final follow-up sample consisted of *N* = 131 participants, indicating a drop-out rate of 64% (baseline sample *N* = 364). In total *n* = 233 participants were lost to follow-up. The majority of drop-outs did not provide consent to be contacted again after initial study participation (*n* = 90), *n* = 64 actively refused participation at follow-up, *n* = 58 could no longer be reached and *n* = 21 patients were confirmed dead. Figure 1 provides a flow chart of the study participants from baseline to follow-up. The follow-up period took place between February 2021 and March 2022 after a mean time to baseline of *M* = 2.70 [2.0–4.0] years. All patients provided written informed consent.

### Assessments

#### Sociodemographic characteristics

Sociodemographic data including gender, date of birth, marital status, employment status, long-term care level,^1^ and degree of disability^2^ were acquired via questionnaire.

#### Mental health state

To assess anxiety and depressive symptoms, the German version of the *Hospital Anxiety and Depression Scale* (HADS-D) was used [24, 25]. The HADS is a self-report questionnaire consisting of a depression and an anxiety subscale. The scales can be considered separately or a total score for overall mental impairment can be generated. Both subscales contain seven items each that are rated on a 4-point Likert scale. An anxiety/depression score between zero and seven is considered not indicative (‘negative’) of anxiety/depression, whereas a score of eight and above indicates the presence (‘positive’) of anxiety/depression. For the total scale, a score of 14 and above indicates the presence (‘positive’) of anxiety/depression. Cronbach’s Alpha for both subscales is 0.80 [25].

Patients’ MD diagnosis status was taken from the baseline study [19, 22, 23]. Diagnosis was expertly assessed based on the *Structured Clinical Interview for DSM-IV* (SCID-I) [26], which was conducted when a patient scored eight or higher on a subscale of the HADS.

#### CHD severity

At baseline, CHD severity was assessed by screening the participants’ medical chart for *New York Heart Association* (*NYHA*) functional classification [27], cardiac events in the previous medical history (e.g., myocardial infarction), cardiac surgery (e.g., bypass surgery), congestive heart failure, somatic comorbidity, percutaneous coronary intervention (PCI) and left ventricular ejection fraction. Based on this information, the treating physician classified the overall severity of the CHD as mild, moderate, or high. There was no follow-up measurement of CHD severity.

#### Status of MD-related health care

To assess patients’ MD-related support, diagnostics and treatment, a self-developed questionnaire was used. Patients could choose to either answer the questions about the care provided by their GP or their outpatient cardiologist. The following categories were addressed: current care/needs (e.g. “Do you also talk to your treating general physician/cardiologist about mental health problems?”), diagnostics (e.g. “Have you had a psychological/psychiatric examination?”), treatment (e.g. “Have you been recommended treatment for your mental health issues?”), access to and barriers of mental health support (e.g. “Have you received sufficient information about the content and accessibility of psychotherapeutic care?”), and utilisation of medical care options (e.g. “How many doctor contacts did you have in the last 4 weeks?”). A proportion of the questions were answered by all patients, while some questions had to be skipped if the content did not apply, e.g., if no mental health problems were present.

### Data analysis

Data analysis was conducted using *IBM SPSS Statistics 28*. The dataset was controlled for outliers, missing and implausible values. No outlier had to be removed. For all analyses, the significance threshold was set at *α* = 0.05.

Sociodemographic, mental health and CHD characteristics of the follow-up sample were examined for baseline and follow-up and additionally compared with those of the drop-outs to analyse differences, using chi-squared tests. Changes in depression and anxiety symptoms over time were analysed using paired samples t-tests with HADS anxiety and depression subscores at baseline and follow-up. The assumptions for paired t-tests were sufficiently met. According to Shapiro-Wilk tests, not all variables were normally distributed, however literature shows that paired t-test are robust against this [28, 29]. To check the robustness of our data, we ran Bayesian paired t-tests on the same variables in *JASP 0.18.1.0*.

To analyse whether CHD patients with or without MD at baseline reported different MD-related health care at follow-up, chi-squared tests were performed. For this purpose, MD was defined by the presence of a diagnosis at baseline as indicated by the SCID-I. The chi-squared test assumptions were met. In cases of expected frequencies below 5, the Fisher’s exact test was used [30]. To check the robustness of the data and due to the small sample size, we ran Bayesian contingency tables in JASP 0.18.1.0 with the same variables.

## Results

### Sociodemographic and mental health characteristics

As shown in Table 1, the mean age at follow-up was 68.32 years and the sample was predominantly male. Most patients lived together with their partner and were retired. The baseline sample was comparable to the follow-up sample in terms of sociodemographic characteristics (see Table 1). Furthermore, no significant differences were found between the follow-up sample and drop-outs with respect to CHD severity, sociodemographic and mental health characteristics at baseline. Refer to Table 2 for a detailed summary.

**Table 1:** Sociodemographic and mental health characteristics of the study sample at baseline and follow-up

	**Baseline**		**Follow-up**	
	***N* = *364, n (%)***	***M* (*SD*)**	***N* = *131, n (%)***	***M* (*SD*)**
**Gender**				
Male	258 (70.9)		94 (71.8)	
Female	106 (29.1)		37 (28.2)	
**Age**		65.88 (11.41)		68.32 (9.66)
35–49 years	24 (6.6)		1 (0.8)	
50–59 years	89 (24.5)		30 (22.9)	
60–69 years	111 (30.5)		43 (32.8)	
70–79 years	90 (24.7)		39 (29.8)	
80–95 years	50 (13.7)		18 (13.7)	
**Marital status**				
Living together	264 (72.5)		99 (75.6)	
Living alone	100 (27.5)		32 (24.4)	
**Employment**				
Full-time	123 (33.8)		27 (21.1)	
Part-time	32 (8.8)		9 (7.0)	
Unemployed	32 (8.8)		16 (12.5)	
Pension	177 (48.63)		76 (59.4)^a^	
**Long-term care level**				
With long-term care level	22 (6.0)		26 (20.0)	
Without long-term care level	342 (94.0)		104 (80.0)^b^	
**Degree of disability**				
With degree of disability	159 (43.7)		76 (58.0)	
No degree of disability	205 (56.3)		55 (42.0)	
**HADS anxiety**		5.38 (3.84)		5.89 (4.24)
Positive (≥ 8)	86 (23.6)		43 (32.8)	
**HADS depression**		4.25 (3.42)		5.77 (4.30)
Positive (≥ 8)	62 (17.0)		45 (34.4)	
**HADS total**		9.63 (6.65)		11.66 (7.88)
Positive (sum score ≥ 14)	91 (25.0)		49 (37.4)	

*HADS* Hospital Anxiety and Depression Scale

A "positive" HADS screening result indicates at least mild symptom severity

^a^*n* = 3 missing values

^b^*n* = 1 missing value

**Table 2:** Sociodemographic characteristics of non-drop-outs and drop-outs

	**Non-drop-outs (= Follow-up)**		**Drop-outs**		***χ***^*2*^	***P*-value**^**a**^	***BF***_***10***_^***b***^
	***N* = *131, n (%)***	***M* (*SD*)**	***N* = 233, *n* (%)**	***M* (*SD*)**			
**Gender**					.267	.606	.140
Male	95 (72.5)		163 (70.0)				
Female	36 (27.5)		70 (30.0)				
**Age**		66.72 (9.73)		65.42 (12.26)			
35–49 years	3 (2.3)		21 (9.0)		9.19	.056	.188
50–59 years	32 (24.4)		57 (24.5)				
60–69 years	46 (35.1)		65 (27.9)				
70–79 years	36 (27.5)		54 (23.2)				
80–95 years	14 (10.7)		36 (15.5)				
**Marital status**					.95	.329	.0193
Living together	99 (75.6)		165 (70.8)				
Living alone	32 (24.4)		68 (29.2)				
**Employmeent**					1.16	.559	.033
Full-time	41 (31.3)		82 (35.2)				
Part-time	10 (7.6)		22 (9.4)				
Unemployed	80 (61.1)		129 (55.4)				
**Long-term care level**					1.79	.181	.150
With long-term care level	5 (3.8)		17 (7.3)				
No long-term care level	126 (96.2)		216 (92.7)				
**Degree of disability**					1.11	.293	.234
With degree of disability	62 (47.3)		97 (41.6)				
No degree of disability	69 (52.7)		136 (58.4)				
**CHD severity**					1.79	.409	.039
Mild	155 (42.6)		98 (42.1)				
Moderate	187 (51.4)		118 (50.6)				
High	22 (6)		17 (7.3)				
**HADS anxiety**		5.63 (4.00)		5.24 (3.75)			
Positive (≥ 8)	35 (26.7)		51 (21.9)		1.08	.298	.200
**HADS depression**		4.37 (3.45)		4.19 (3.41)			
Positive (≥ 8)	26 (19.8)		36 (15.5)		1.15	.284	.184
**HADS total**		10.0 (6.80)		9.43 (6.57)			
Positive (sum score ≥ 14)	36 (27.5)		55 (23.6)		.67	.412	.166
**SCID-I diagnosis**	42 (32.1)^b^		60 (25.8)^c^		1.66	.198	.280
Affective disorder	22 (52.38)		29 (48.33)				
Phobia	9 (21.43)		10 (16.67)				
Posttraumatic stress disorder	5 (11.9)		8 (13.33)				
Substance use disorder	2 (4.76)		5 (8.33)				

Data is derived from baseline timepoint

HADS Hospital Anxiety and Depression Scale, SCID-I Structured Clinical Interview for DSM-IV

A “positive” HADS screening result indicates at least mild symptom severity

^a^In cases of expected frequencies below 5, the Fisher’s exact test was used

^b^For all tests, the alternative hypothesis specifies that group drop-outs is not equal to non-drop-outs. The sample was set on independent multinomial, columns fixed

^c^*n* = 4 specific diagnosis missing

^d^*n* = 8 specific diagnosis missing

In terms of mental health, about one-third of the follow-up sample had an MD diagnosed based on the SKID-1 at baseline. The majority of these patients were diagnosed with an MD belonging to the spectrum of affective disorders (see Table 2). This distribution was reflected in the HADS scores of the follow-up sample. For each subscale (total, anxiety, and depression), the mean score in the sample was below the cut-off for clinically relevant symptoms (≥ 8 for HADS depression and anxiety, ≥ 14 for HADS total), while about a third of patients scored above the cut-off, indicating the presence of at least mild symptoms. Both Bayesian and classical analyses showed that in the non-MD patient group, HADS scores for the depression subscale and the total scale increased significantly from baseline to follow-up, indicating a decline in mental health (see Table 3). For the group of MD patients, the HADS scores did not show significant differences from baseline to follow-up (see Table 3).

**Table 3:** Course of anxiety and depression symptoms from baseline to follow-up

	**t**	***P***	**BF**_**10**_^**a**^	**Error %**
**Non-MD (n,%)** 89 (67.94)				
**HADS anxiety**	-1.714	.090	.476	.042
**HADS depression**	-4.973	**< .001**	**4718.195**	1.326 × 10^–10^
**HADS total**	-3.736	**< .001**	**63.525**	2.851 × 10^–8^
**MD (n,%)** 42 (32.06)				
**HADS anxiety**	.649	.520	.203	.051
**HADS depression**	-1.209	.234	.329	.042
**HADS total**	-.276	.784	.173	.054

Cauchy scale was set at 0.707

^a^For all tests, the alternative hypothesis specifies that measure one and measure two are not equal

### Status of MD-related health care in relation to the presence of MDs

Significant differences were found in the following areas of MD-related health care: more MD patients than non-MD patients received a psychological/psychiatric examination, an MD diagnosis and a treatment recommendation for mental health problems. Additionally, the physician was more responsive to the request for psychotherapy in MD compared to non-MD patients. Small, but non-significant differences were found in the following areas: MD patients talked to their physician about mental health problems more frequently, and they were more likely to know about the result of their MD diagnostics, to receive help in search for psychotherapeutic treatment and to feel adequately informed regarding psychotherapy and psychopharmacotherapy in comparison to non-MD patients. Furthermore, more MD patients than non-MD patients reported currently receiving psychotherapy. No differences in MD-related health care between non-MD and MD patients were found concerning the physicians’ active demand for patients’ mental health, referral to a specialist for additional diagnostics, response to the request for psychopharmacotherapy, help received in search for psychopharmacotherapy and currently undergoing psychopharmacotherapy. For all tests, both Bayesian and classical analyses led to the same conclusions, underlining the robustness of our analyses. Detailed results are presented in Table 4.

**Table 4:** Follow-up state of MD-related health care

	**Non-MD** ***n (*%)**	**MD** ***n (*%)**	***χ***^***2***^	***P*-value**^**a**^	**φ**	***BF***_***10***_^***b***^
Talking with physician about mental health problems	37 (42.0)	24 (57.1)	2.60	.107	.14	.829
Active demand for mental health problems by physician	23 (46.0)	12 (41.4)	.16	.690	-.05	.304
Found it appropriate to have been asked by physician	26 (57.8)	11 (44.0)	1.22	.269	-.13	.534
Psychological/psychiatric examination carried out	**6 (16.7)**	**19 (55.9)**	**11.71**	**< .001**	**.41**	**97.909**
Diagnosed positively mental disorder symptoms	**5 (13.5)**	**13 (38.2)**	**5.43**	**.020**	**.28**	**2.790**
Referral for additional diagnostics	5 (13.5)	6 (17.6)	.23	.631	.06	.262
Patient’s knowledge about mental disorder diagnosis	12 (33.3)	18 (52.9)	2.75	.098	.20	.689
Wish for treatment for mental health problems	10 (29.4)	14 (45.2)	1.73	.189	.16	.599
Recommendation for treatment of mental health problems	**4 (11.8)**	**12 (38.7)**	**6.34**	**.012**	**.31**	**4.977**
Currently undergoing psychotherapy	3 (8.8)	8 (25.8)	3.33	.068	.23	.700
Currently undergoing psychopharmacotherapy	5 (14.7)	7 (23.3)	.78	.378	.11	.315
Received sufficient information about the content and/or accessibility of psychotherapeutic treatment	6 (18.8)	10 (32.3)	1.52	.218	.16	.447
Treating physician responded to request for psychotherapeutic treatment	**5 (16.7)**	**13 (41.9)**	**4.68**	**.031**	**.28**	**3.182**
Received help in search for psychotherapeutic treatment	2 (6.7)	8 (25.8)	4.01	.081	.26	1.271
Received sufficient information about the content and/or accessibility psychopharmacotherapy	6 (20.0)	12 (37.5)	2.30	.129	.19	.453
Treating physician responded to request for psychopharmacotherapy	7 (22.6)	7 (22.6)	.00	1.000	.00	.469
Received help in search for psychopharmacotherapy	6 (20.0)	8 (25.8)	.29	.590	.07	.443

Significant results are shown in bold. Due to the construction of the questionnaire, patients skipped questions that did not apply to them. Therefore, the sample size varies between questions

*MD* Mental disorder - defined by the presence of a diagnosis at baseline as indicated by the SCID-I

^a^In cases of expected frequencies below 5, the Fisher’s exact test was used

^b^For all tests, the alternative hypothesis specifies that the two groups are not equal. The sample was set on independent multinomial, columns fixed

## Discussion

This study aimed to assess differences in MD-related health care among CHD patients with and without MD, who were diagnosed 2–4 years prior at baseline. We found that MD-related health care at follow-up differed in four aspects of care between CHD patients with and without MD at baseline: MD patients were examined psychologically/psychiatrically and diagnosed with MD by their GP or cardiologist more frequently, a treatment recommendation was given more often to MD patients and the physician was more responsive to their request for psychotherapeutic treatment. There were no significant differences between the treatment of patients with and without MD concerning the physicians’ active demand for patients’ mental health, the referral to a specialist for additional diagnostics, the provided information about the diagnosed MD and further treatment options, the response to the patient’s request for psychopharmacotherapy, help received in finding psychotherapy or psychopharmacotherapy, and actually receiving these treatments.

Although not statistically significant, we found a trend that more MD than non-MD patients were currently receiving psychotherapy. In addition, small, non-significant differences were found with regard to communication with the physician about mental health problems, adequate information regarding psychotherapeutic and psychopharmacotherapy and received help in search for psychotherapeutic treatment. These visible but non-significant differences between the treatment of MD and non-MD patients might at least partly be explained by the low sample sizes and thus, insufficient power to detect differences between groups. The results partially confirm our hypothesis as MD-related treatment differs in terms of diagnostics and recommendation for further treatment, especially regarding psychotherapy, but the GP or cardiologist gives little support or information about further diagnostics or finding adequate treatment for those diagnosed with MD.

In line with previous research [18, 19, 23], our results support the assumption that MD-related care for CHD patients with comorbid MD is insufficient. Despite the fact that more MD patients were screened for and positively diagnosed with an MD than non-MD patients, our baseline study [19] showed a detection rate of only 48.0%, which is unsatisfying. Furthermore, despite guidelines emphasise the need for special attention and support for CHD patients with MD, such as additional treatments like psychotherapy or psychopharmacotherapy [6], in our study almost 60% of MD patients did not receive a treatment recommendation for their mental health problems and approximately 75% did not receive support in their search for psychotherapy/psychopharmacotherapy. Our results are comparable to the research of Kuhlmann et al. [18] showing that the majority of MD patients did not receive any MD-related treatment or treatment recommendations. Overall, the findings suggest that recommended routine screening and adequate treatment for comorbid MD in CHD patients is not being implemented comprehensively.

Various explanations for the insufficient MD-related health care in CHD patients are conceivable. Lack of knowledge about the relevance of comorbid MD in CHD [6, 14, 15] may play a role. This may also depend on the type of MD, as Westermair et al. [20] showed that the majority of CHD patients with comorbid depression received MD-related treatment, whereas there were deficits in diagnosis and treatment of comorbid anxiety disorders. Lack of trust in mental health care and stigmatising attitudes towards mental health on the part of physicians may also be involved [31]. The latter in particular can strain the patient-physician relationship, tending to discourage open communication about mental health problems [31]. In this context, trainings, e.g. in psychosomatic care and person-centered communication, might be helpful to increase an empathic communication and expertise in comorbid MD [32, 33]. Of course, structural problems may also play a role in the insufficient health care of CHD patients with comorbid MD, such as lack of time and insufficient compensation for additional workload due to screening procedures and complex conversations [12].

### Strengths and limitations

To our knowledge, this study is unique in that it follows patients with CHD from different clinical settings, who are well-characterised and have received in-depth assessment of potential MDs, allowing observation of their mental health and MD-related care over time. Study participation provided previously undiagnosed MD patients with a validated diagnosis and the opportunity to receive treatment. In addition, health care is described from the patient’s perspective, which allows to take individual wishes, needs and perceptions of patients into account.

Nevertheless, there are limitations to our study. The self-report measurements that were used in this study, despite their aforementioned advantages, are susceptible to response biases, such as social desirability or recall bias. Additionally, due to the high drop-out rate, the present study sample was rather small, which limits the validity and generalizability of the observed results. However, there was no evidence of systematic drop-out reasons, as those patients who participated at follow-up and those who dropped out did not differ in sociodemographic and mental health characteristics or CHD disease severity at baseline.

Furthermore, since the corona pandemic took place between baseline and follow-up measurement, mental health and health care characteristics may have been confounded by a general increase in anxiety and depression [34] and a decrease in the use of medical services due to protective measures [35].

### Implications for further research

While our sample included patients from different health care institutions, most of them were living in the urban region of Cologne, Germany. Further studies focusing on primary health care for CHD patients in rural areas would be relevant to analyse potential differences between these settings. Additionally, a longer follow-up time and frequent study visits would be beneficial to better understand the factors that influence the course of MD symptoms in this patient group.

As highlighted before, recommendations to improve care through trainings in basic psychosomatic care and patient-centered communication [32, 33] have been published. In addition to general approaches, specific interventions for the detection and treatment of MD in CHD could be successful. As part of the MenDis-CHD project, a pilot intervention was developed, which specifically addresses the identification and management of mental and cognitive complaints of CHD patients in the primary care setting [27]. These findings may offer insights into the feasibility of specific interventions for physicians treating CHD, while providing an impetus for further development and expansion of such interventions.

## Conclusion

In this prospective cohort study, we observed that the detection of MDs and MD-related health care for CHD patients is largely not in line with guideline recommendations. These findings highlight the need to raise awareness among physicians to address mental health in CHD patients in an empathetic and supportive way. Targeted detection, e.g., through routine screening for MD symptoms, should be encouraged to generate a potentially positive impact on the health care and health outcomes of patients with CHD.

## Data Availability

The dataset supporting the conclusion of this article cannot be shared publicly because of ethical restrictions imposed by the Ethics Commission of Cologne University’s Faculty of Medicine. Data access queries may be directed to the CoRe-Net Coordination Team of the Institute of Medical Sociology, Health Services Research and Rehabilitation Science (IMVR) (email: Core-Net@uk-koeln.de) or to Dr. Peter Ihle, CIO of data management and data protection of the PMV research group of the Cologne University (contact via tel.: +49 221 478 85532 or via email: peter.ihle@uk-koeln.de).

## Abbreviations

BMBF: Bundesministerium für Bildung und Forschung (English: Federal Ministry of Education and Research)
CECAD: Cluster of Excellence Cellular Stress Responses in Aging-Associated Diseases
CHD: Coronary heart disease
CoRe-Net: Cologne Research and Development Network
DZNE: Deutsches Zentrum für neurodegenerative Erkrankungen (English: German Center for Neurodegenerative Diseases)
GP: General practitioner
GdB: Grad der Behinderung (English: degree of disability)
HADS-D: Hospital Anxiety and Depression Scale- Deutsche Version (English: Hospital Anxiety and Depression Scale- German version)
ISS: Institut für Soziolgie und Sozialpsychologie (English: Institute of Sociology and Social Psychology)
MD: Mental disorder
MDK: Medizinischer Dienst der Krankenkasse (English: Health Insurance Medical Service)
MenDis-CHD: Mental Disorders in coronary heart disease
SCID-I: Structured Clinical Interview for DSM-IV
ZVFK: Zentrum für Versorgungsforschung Köln (English: Centre for Health Services Research Cologne)

## Footnotes

1 The Health Insurance Medical Service (MDK) determines health impairments of autonomous living and abilities in different areas of life, resulting in one of five levels of impairment: minor, considerable, serious, severe, most severe. Each level is associated with a certain amount of financial and/or nursing-services support.

2 Degree of disability (Grad der Behinderung; GdB) assessed by the pension office, indicates how severely a person is affected by their disease or disability. It is expressed in degrees of 10 (lowest = 20, highest = 100). The higher the value, the more severe the disability; from a GdB of 50, a person is considered severely disabled.
